# Separate requirements for detection and perceptual stability of motion in interocular suppression

**DOI:** 10.1038/s41598-017-07805-5

**Published:** 2017-08-03

**Authors:** Egor Ananyev, Trevor B. Penney, Po-Jang (Brown) Hsieh

**Affiliations:** 1 Department of Psychology, National University of Singapore, Singapore, Singapore; 2 LSI Programme in Neurobiology and Aging, National University of Singapore, Singapore, Singapore; 3 Neuroscience and Behavioral Disorders Program, Duke-NUS Medical School, Singapore, Singapore

## Abstract

In interocular masking, a stimulus presented to one eye (the mask) is made stronger in order to suppress from awareness the target stimulus presented to the other eye. We investigated whether matching the features of the target and the mask would lead to more effective suppression (feature-selective suppression), or not (i.e., non-selective suppression). To control the temporal characteristics of the stimuli, we used a dynamic interocular mask to suppress a moving target, and found that neither matching speed nor pattern of motion led to more effective suppression. Instead, a faster target was detected faster, regardless of the mask type or speed, while a relatively slow (about 1°/s) mask was more perceptually stable (i.e., maintained suppression longer) in a non-selective fashion. While the requirement for target detectability, i.e., salience, is well characterized, relatively little attention is given to the factors that make a mask percept more perceptually stable. Based on these results, we argue that there are separate requirements for detection and perceptual stability.

## Introduction

When the two eyes are presented with conflicting images of equal perceptual strength, instead of fusing the two, awareness may switch repeatedly and stochastically from one image to the other over time. This phenomenon, termed binocular rivalry, presents a way to study unconscious processing because the observer is intermittently unaware of one of the image despite its continuous presence^1, 2^. Stochastic switches, however, make it difficult to study the time course of rivalry, and this drove development of other techniques, such as continuous flash suppression (CFS)^3^. CFS allows for the target stimulus to be suppressed for much longer durations, and led to a multitude of findings on the extent of unconscious processing in perceptual after-effects^3^, salience^4^, scene and face perception^5–10^, decision-making^11^, working memory^12–14^, numeric and language processing^15–18^, among other topics.

A link between CFS and binocular rivalry was established at the inception of the CFS technique^3^, so insights gained from the binocular rivalry research suggest possible mechanisms behind the effectiveness of CFS. One such idea is *resistance to adaptation* of the mask. In binocular rivalry, adaptation is presumed to contribute to the eventual replacement of the dominant image by the suppressed one^19^. In the original CFS publication, Tsuchiya and Koch (2005) suggested that it is perhaps the resistance to adaptation of the mask that leads to its effectiveness, while the rapid updating of the mask’s images ensures its continual dominance.

A complementary explanation is called *feature-selective suppression*. The idea is that the features of the target that are represented in the mask are more effectively suppressed^20, 21^. Sometimes, the feature-selective suppression concept is used by researchers to guide the creation of novel masks that more closely match the surface characteristics of the target^22^. For example, a CFS mask composed of more circular features, as opposed to a more traditional rectangular Mondrian mask, led to more optimal suppression of faces^23^, while a rectangular CFS mask more effectively suppressed noise patterns that were band-pass filtered either vertically or horizontally^24^. Similar effects obtain for binocular rivalry: a probe that did not match the oblique orientations of two rival gratings was more easily detected^21, 25^ and a colored target was best suppressed with a colorful mask, while using a grey scale mask resulted in “leakage” of the target color into the mask elements, with the target shape still being suppressed^26^.

Yet there are a few indications of a fundamental asymmetry in the relationship between the features of the mask and the target. For example, even though a target low in spatial frequency was best suppressed by a matching mask, a high spatial frequency target was poorly suppressed by masks of any spatial frequency^24^. In the same vein, an interocular mask with a specific temporal frequency was most effective for suppressing static stimuli^3, 24^. Tsuchiya and Koch^3^ (supplemental materials) observed that a CFS updating frequency from 3–12 Hz led to optimal suppression (hence the popular use of 10 Hz in many studies). At the same time, traditional CFS is relatively poor at suppressing motion^27^, while observers are more sensitive to linear motion as opposed to more complex patterns (e.g., rotational) under binocular rivalry suppression^28, 29^, revealing that targets, too, have a set of distinct characteristics that are optimal for detection, namely salience. These observations suggest a characterization of binocular suppression as generally a non-selective process^30^, with feature-selective suppression merely improving the already established dominance of the interocular mask via a set of specific features, such as high contrast, specific temporal and spatial frequency, etc., that are independent of the target.

These observations point to a preferred mask configuration that is optimally effective for suppressing a range of target features, which in turn suggests the existence of a dimension characterizing the *perceptual stability* of a mask, — or any stimulus, for that matter — in an interocular competition. Viewed in this light, in addition to providing a time course and mechanism for manipulating target transition from preconscious to conscious awareness, the interocular masking paradigm is a potential tool for evaluating the ability of a stimulus (mask) to maintain its conscious dominance, thereby linking the interocular masking literature to the larger body of work on binocular rivalry, specifically, predominance^28, 31, 32^.

If perceptual stability is conceived of as a dimension that benefits from certain stimulus characteristics, motion is the prime suspect. Utilizing motion as a mask in binocular rivalry is far from new^1, 3, 33–35^ due to its potency of suppression^27^. One vivid example of the power of motion in dominating our awareness is the so-called Cheshire Cat Effect^36^. With one hand holding a mirror in front of one eye, the observer moves her other hand in the reflection, while looking at someone else’s smiling face with the other eye. For most observers, this leads to the moving hand suppressing the face either entirely, or in patches, such that only some features, such as eyes and teeth, remain visible (hence the name of the illusion).

Van de Grind *et al*.^37^ found that two brief and sparse binocularly presented dot patterns similar in speed were likely to result in rivalry, implying that feature-selectivity may apply to movement. However, if the speeds were disparate enough, the two moving patterns instead overlapped in space, creating the illusion of transparency. This led the authors to suggest separable motion channels for fast and slow movement, and that binocular rivalry is primarily caused by within-channel competition. However, this finding is challenged by evidence that certain binocular rivalry scenarios cause more rivalry overall. For instance, slower speed caused rivalry more often than higher speed^38^. If rivalry were simply a matter of within-channel competition, why would some channels show more rivalry than others?

Given these considerations, we employed a dynamic mask with a moving target to evaluate whether motion follows the feature-selective suppression hypothesis, with the most effective mask matching the motion characteristics of the target. An alternative outcome is that the dynamic mask has requirements for optimal suppression that are distinct from the characteristics of the target movement. The target and mask speeds and the movement patterns (linear/rotational) were manipulated. If the feature-selective hypothesis applies to motion, matching the mask speed and pattern of motion with those of the target will lead to the most effective suppression. Alternatively, the perceptual stability of the mask may benefit from motion patterns and speeds that are not tied to those of the target. The intention was to evaluate the feature-selective hypothesis with regards to motion, and specifically, velocity and pattern of movement, to build a more complete picture of the relationship between target and mask characteristics in interocular suppression.

To preview the results, we found that whether the target broke suppression sooner or the mask maintained its dominance longer was primarily decided by their respective speeds and motion patterns. Furthermore, increasing the mask speed past a relatively low velocity (1–2°/s) rendered it less effective, regardless of the target speed, while faster targets broke suppression faster, regardless of the mask speed. From this we conclude that the feature-selective hypothesis does not hold for interocular masking of movement, and propose instead that perceptual stability depends on a set of requirements that are quite distinct from salience, and is potentially influenced by the organization of the visual pathways.

## Experiment 1

There were two primary objectives for the first experiment: (1) evaluate the influence of mask speed on its effectiveness; (2) examine the effect of the pattern of motion on breaking suppression.

A previous study by Moors *et al*. (2014) found that matching the dynamic mask’s speed to that of a moving target provided the most effective suppression. The hypothesis was therefore that there would be a peak in suppression times for the mask moving at 3°/s, corresponding to the speed of the target ― consistent with feature-selective suppression.

Some research on binocular rivalry also suggests that the pattern of movement matters: linear movement is more fully processed under suppression than complex motion patterns, such as rotating or spiral motion. Sensitivity to complex motion was reduced under suppression^28, 29^. An early study also found that a translational stimulus was detected faster than a rotating stimulus^34^, but linear motion was confounded with higher velocities, such that no strong conclusions could be drawn.

If the feature-selective hypothesis holds, we would expect that matching speeds and patterns of motion of the target and the mask would lead to more effective suppression. On the other hand, if mask and target have divergent criteria for maintaining dominance and detection, respectively, we would expect (1) a specific mask speed to be most effective, with higher or lower speeds leading to less effective suppression, and (2) consistent with past research, a linearly moving target would break suppression faster, while the pattern of motion of the mask would not matter.

### Results

**Figure 1:**
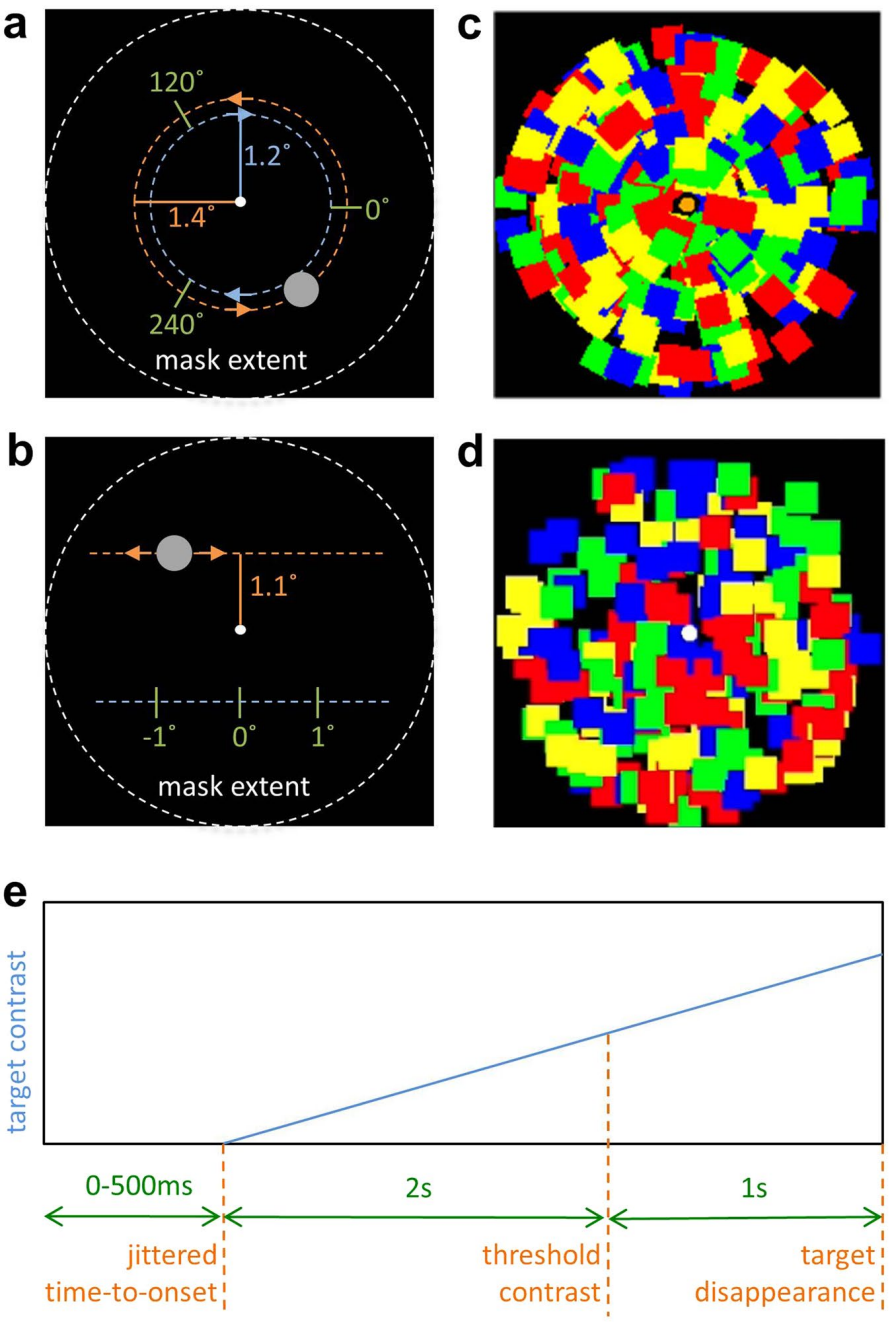
Target and mask configurations and target contrast ramp-up. (**a**,**b**) Target configuration. Target in *grey*; *green*: starting positions; *orange and blue*: movement direction, possible trajectories and their distances from the center; *white*: the extent of the mask. (**a)** Rotational target. (**b)** Linear target.(**c**) Rotational mask. (**d)** Linear mask. (**e**) A schematic of change in target contrast throughout the trial. The target begins to accrue in contrast after a jittered onset (0–500ms) and reaches the contrast threshold (variable during threshold measurement and fixed during the main experiment) at 2 s. The participant’s task is to press a button as soon as the target is detected. The target always disappeared 3 s after the onset, even if the button was already pressed, to discourage speeded responses. If the threshold value was greater than 1 (i.e., the target was too difficult to detect even with a ramp up to maximum value within 2 s), the contrast was ramped up to maximum (1) at 2 s and remained there for the rest of the trial, while the mask contrast was reduced by (2 – threshold) and did not vary in a given trial.

The target and the mask either rotated or moved linearly (Fig. 1), such that their pattern of motion matched or did not, and the participants were asked to indicate as soon as they detected the target that was ramped up in contrast (Fig. 1E). The speed of rotating target and mask were computed in terms of linear, and not rotational, velocities, so that rotating and linear pattern of motions are comparable (see **Methods** for more details). Linear targets and mask elements repeated their movement trajectories when the edge was reached, so that they also moved in a cyclical fashion similar to the rotating targets and mask elements.

There was high variability in thresholds among participants (mean threshold = 1.24; *SD* = 0.52). Seven out of 11 participants required decreased mask contrast for the target presented at full contrast to be visible. This suggests that the mask was highly effective in suppressing the moving target. A similar pattern was found for Experiments 2 and 3.

False alarms were relatively rare: fewer than 20% of trials across participants and fewer than 5% for all but two participants.

**Figure 2:**
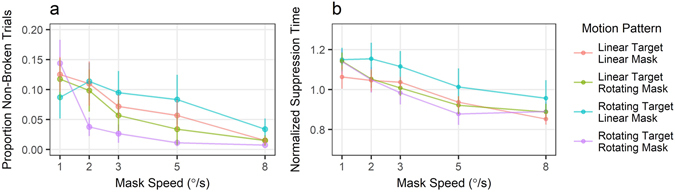
The relationship between the mask speed and target/mask motion patterns in Experiment 1. (**a**) Proportion of non-broken (no-response) trials. Although there was a tendency for the rotating mask to be less effective, there was no effect of matching target and mask motion pattern. (**b**) Suppression times normalized by subject means across conditions. Dots are group means with error bars reflecting standard errors.

A linear mixed effects model (with participant ID as a random factor) was used to test the fixed effects of mask speed and matching motion patterns between mask and target on the number of trials that did not break suppression (Fig. 2A) and suppression times in those that did (Fig. 2B). To evaluate the effect of mask speed, it was centered at 3°/s (the speed of the target). A binary variable indicated a match between the motion patterns of the target and the mask, with ‘one’ if both were moving linearly / rotationally, ‘zero’ otherwise.

Higher mask speeds resulted in less effective masking of the target, as indexed by a decrease in the number of no-response trials (i.e., when the target was not detected; Fig. 2A). Increasing mask speed led to fewer such trials (*b* = −0.290, *t* = −6.12, *p* <0.001). Matching the mask pattern of motion to that of the target did not have an effect on the number of no-response trials (*b* = −0.121, *t* = −0.98, *p* = 0.329).

Similar effects were found for the effects of mask characteristics on suppression times (Fig. 2B). Faster masks were less effective at suppressing the target, with every 1°/s increase in mask speed reducing the detection time by an additional 50 ms (*t* = −15.90, *p* <0.001). Motion pattern matching produced an effect, but in the opposite direction from what would be expected based on the feature-selective suppression, as matching mask and target motion patterns led to *shorter* detection times (*b* = −0.025, *t* = −2.91, *p* = 0.004). As can be seen in Fig. 2B, this is primarily due to the marginally more effective suppression of linearly moving masks, as indexed by the proportion of non-broken trials and suppression length (*p* = 0.078 and *p* = 0.042, respectively). However, Bayesian statistics show no conclusive evidence of the contribution of the mask pattern to the two models compared to partial models (*BF* = 1.02 and *BF* = 6.86, respectively).

### Discussion

While matching the pattern of motion of the mask to that of the target had no effect on target detection, the speed of the mask had a clear effect on its effectiveness. Perhaps counter-intuitively, the slower masks led to far more stable suppression, with speeds of 1 and 2°/s leading to highest target detection times, while the mask matching the speed of the target (3°/s) did not lead to optimal suppression, which is counter to the feature-selective hypothesis. There was a marginal effect of mask motion pattern on suppression effectiveness, with rotating masks being slightly less effective than linearly moving ones. In addition, there was no relationship between the target motion pattern and its detection time. These effects were largely mirrored by the contrast sensitivity measurements under interocular suppression (see Supplementary Figure S1).

Although there is no evidence for the feature-selective hypothesis with regards to the motion pattern, there was a slight increase in suppression duration of a rotating target by a linear mask, which was more apparent for subjects with higher thresholds (i.e., for whom mask contrast was reduced in order for them to be able to detect the target; see Supplementary Figure S2). It is possible that this was due to subthreshold summation, with the low mask contrasts actually facilitating target detection, as observed with pedestal masking paradigms^39^. This would explain why the two matching motion pattern scenarios led to faster detection only for high-threshold subjects; however, this does not explain why the same thing happened for the other non-matching scenario, i.e., linear target and rotating mask.

## Experiment 2

One possible explanation for the effect of the mask speed on suppression effectiveness is that it is the *difference* between the mask and target speed that leads to a specific pattern of suppression effectiveness, rather than the mask speed per se. In other words, based on the results of the first experiment, it is possible, for instance, that a mask has to be ~2°/s slower than the target for optimal suppression, perhaps due to the size, shape, or other feature differences between the target and the mask. To address this possibility, in the second experiment, the target’s motion was either fast (5°/s) or slow (1°/s). It was hypothesized that higher target speeds would lead to shorter detection times, regardless of the mask speed. Additionally, we introduced a lower mask speed (0.3°/s) to test whether there was a decrease in suppression effectiveness for an extremely slowly moving mask.

As the motion patterns did not have a dramatic effect on suppression effectiveness in Experiment 1, and to eliminate the contribution of transients associated with the periodic resetting in the linear motion condition, both the mask and the target moved rotationally in Experiment 2.

### Results

**Figure 3:**
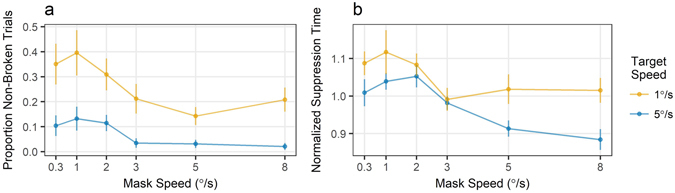
The relationship between the target and mask speeds and the suppression strength in Experiment 2. (**a**) Proportion of no-response trials. The faster targets were more consistently detected. (**b**) Faster targets were also detected faster. Matching the mask speed to that of the target did not lead to most effective suppression: the optimal mask speed was approximately the same (1–2°/s) for both target speeds. Dots are group means with error bars reflecting standard errors.

As shown in Fig. 3, and consistent with Experiment 1, there was a main effect of mask speed, with slower masks producing more effective suppression, as indexed by both the no-response trials and suppression times (*b* = –2.308, *t* = −4.38, *p* <0.001 and *b* = –0.022, *t* = −21.68, *p* <0.001, respectively). The effect of target speed was also significant, with the faster target detected more consistently and faster compared to the slower target (*b* = –4.651, *t* = −6.73, *p* <0.001 and *b* = –0.022, *t* = −14.71, *p* <0.001, respectively). There was a small, but significant interaction for the suppression times (*b* = –0.003, *t* = −6.50, *p* <0.001), but not for the number of no-response trials (*p*> 0.4).

There was also a significant quadratic relationship between mask speed and the suppression times (*F*[1, 4] = 16.48, *p* = 0.015). The longest suppression for both slow and fast targets was achieved by a mask moving at about 1°/s, as the slope changed from positive at mask speeds 0.3–1°/s (*b* = 0.042, *t* = 3.03, *p* = 0.002) to negative at mask speeds 2–3°/s (*b* = −0.035, *t* = −3.69, *p* <0.001). For both of these contrasts, there was no interaction of with the target speed (*p*-values> 0.05).

### Discussion

Faster targets were detected faster, which is consistent with the salience hypothesis and the well-established effect of velocity on contrast threshold e.g., ref. 40. However, the absence of a similar speed effect on the effectiveness of the mask suppression (faster masks were in fact less effective) suggests a different mechanism for perceptual stability, and is addressed in full in the General Discussion section.

In addition, we again found that matching the mask and target speeds did not lead to the most effective suppression, contrary to the feature-selective hypothesis. For both target speeds, the most effective suppression was achieved by a mask moving at 1–2°/s, while masks moving faster or slower provided weaker suppression for both target speeds.

The temporal frequency content of the masks and targets moving at different velocities was also analyzed (for rotational pattern of motion), and it did not alter the conclusions made based on velocities: increasing the velocity expanded the spectral profile of temporal frequencies to include those at the higher end of the spectrum in a similar fashion for both target and mask, and no evidence of feature-selective suppression emerged (Supplementary Figure S4).

## Experiment 3

Given the periodic target trajectories in previous experiments, the faster targets might have repeated a part of the trajectory, while the slower ones did not. A question that arises is what characteristics of the target speed make it more readily detectable: is it the rate of travel per se, or the number of cycles travelled by the target leading up to detection? This is particularly important in light of the potential role of the retinal adaptation in interocular detection. To address this question, in Experiment 3, we tied the length of the trajectory to the target speed, such that the slower 1°/s target has a fifth of the trajectory length of the faster 5°/s target. The rotating mask was used. If the difference in target speed leads to the same detection pattern as in Experiment 2 (i.e., faster detection of the faster target) when the trajectory length is controlled, we can conclude that the rate of translation *per se* contributes to the target’s detectability. If, on the other hand, we no longer observe such a pattern, and the target speed no longer plays a role, this would suggest that it is the number of cycles travelled (i.e., the number of times a given retinal location is traversed) by a target that contributes solely to its detectability.

### Results

**Figure 4:**
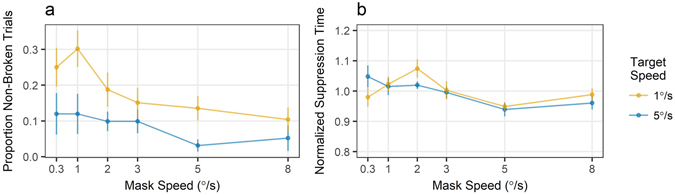
The relationship between the target and mask speeds, and suppression effectiveness in Experiment 3. The distance targets traveled on each iteration was tied to their velocity. (**a**) Faster targets were more consistently detected. (**b**) The effect of the target speed on detection times was no longer significant. Dots are group means with error bars reflecting standard errors.

Once the distance travelled by the target was tied to its speed, and the number of iterations equated between the two target speeds, the faster targets were detected more consistently (*b* = −4.285, *t* = −4.59, *p* < 0.001), yet there was no longer a significant association between the faster targets and shorter detection times (*b* = −0.002, *t* = −1.14, *p* = 0.254; *BF* = 0.039; Fig. 4). As in previous experiments, faster masks were less effective at suppressing a target overall (*b* = −3.022, *t* = −4.24, *p* < 0.001 and *b* = −0.012, *t* = −7.73, *p* <0.001 for no-response trials and suppression times, respectively). Interaction between target and mask speeds did not reach significance (*b = *0.652, *t* = 1.83, *p* = 0.070; *BF* = 1.04 and *b* <0.001, *t* = 0.28, *p* = 0.783; *BF* = 0.027). These results closely resemble those obtained when the target travelled a linear path (Supplementary Figure S3).

### Discussion

Given that the faster targets were more likely to repeat a part of their trajectory, it was important to evaluate which aspect of velocity contributed to target detection. Equating the number of reversals made by both target speeds resulted in more frequent detection of faster targets, yet abolished their advantage in terms of shorter detection times. This divergence between the two indices of suppression effectiveness is notable, and suggests that they either assess different aspects of target detection in the interocular scenario, or detection frequency is more sensitive to mask effectiveness. Nevertheless, we can conclude that the rate of translation *per se* contributes to the target detection.

## General Discussion

The current study showed that neither matching speed nor motion pattern led to the most effective suppression, which contradicts the feature-selective suppression hypothesis. Linearly moving masks with velocity of 1°/s were most effective regardless of target speed or type of motion. This finding also contradicts the retinal adaptation hypothesis of suppression effectiveness, as higher speeds would be expected to lead to greater resistance to retinal adaptation^27^.

These results add to the accumulating evidence suggesting that feature-selective suppression may not apply to all features equally^24, 28–30^. This is not to imply that feature-selective suppression does not occur for certain features, such as shape and color^41^. Indeed, in the current study, these two surface features were intentionally made different between the target and the mask to make the detection of the target easier ― or even possible. Nevertheless, given the importance of motion in interocular suppression^3, 27^, the current study adds to the thesis that certain stimulus features have independent effects on target and mask. Matching the complexity (Experiment 1) or speed (Experiment 2) of target and mask motion did not lead to the optimal suppression. In fact, a mask matching the target motion pattern led to a modest, yet significant, reduction in target detection only (Experiment 1). This is surprising considering the many instances when matching the features of the mask and the target improved suppression effectiveness^21, 23–26^. The evidence against the feature-selective hypothesis may also seem to contradict a recent study by Moors *et al*.^27^, who, despite employing a similar design, found that matching the mask speed to that of the target led to the optimal suppression. However, that pattern of results broke down at higher target speeds in their study; in fact, for all three target speeds tested (1, 3, and 5°/s), the optimal mask speed seems to be similar, i.e., 2–3°/s. Importantly, the current result is not peculiar to the specific paradigm used, as it is consistent with recent evidence of a remarkably slow noise mask for optimal interocular suppression^40^.

On the other hand, the most effective mask speed of 1–2°/s reported here was shown by Blake *et al*.’s finding^28^ to lead to slowest alternations between rival motions, consistent with Levelt’s fourth proposition^2^, which predicts faster alternation for stronger stimuli. In this respect, this agrees with the notion that the slow mask that led to the most effective suppression is not what would traditionally be called a “strong” (or salient) stimulus, and that it is in fact the stimuli that lead to slowest alternation that may lead to most effective suppression. Low-velocity gratings were also tested by Wade, de Weert, & Swanston^32^, who found that increasing the speed of a rival grating up to 0.41°/s led to increased predominance against a static, orthogonally oriented grating (consistent with the increase in slope for speeds 0.3–1°/s in Experiment 2), which implies that a certain minimal stimulus strength is required for optimal suppression, consistent with physiological evidence that V1 cells are tuned to low velocities^42^. In addition, Masson *et al*.^43^ found highest motion segmentation performance at 1°/s, while speed discrimination performance improved with faster velocities. These findings hint at potential stimulus characteristics that are fundamentally different from those that would make for a salient target.

In line with this evidence, one possible explanation for why a certain relatively low-velocity pattern may lead to the most perceptually stable and effective mask may lie in the organization of the early visual pathways^44^. A convention in the use of interocular masks has been to pair a low-contrast, achromatic target with a high-contrast, quickly changing, and typically colorful mask. These properties are meant to both maximize the effectiveness of the mask and minimize the detectability of the target. It can be argued that precisely these characteristics bias the target to rely on the magnocellular (M) pathway, and the mask on the parvocellular (P) pathway processing^45–48^. The M-pathway has a bias toward high temporal and low spatial frequencies, low luminance contrast, and is color-blind; the P-pathway is color-opponent, and operates mostly in low temporal and high spatial frequencies, and high luminance contrast. This separation is not absolute, as some P-pathway neurons are sensitive to motion, and some M-pathway neurons to wavelength. However, the division of labor remains remarkably intact as the two pathways diverge into ventral and dorsal networks, respectively^46, 48–50^.

It is possible that the mask/target visibility status biases them toward a specific pathway: the mask is already visible, so it would rely on the P-pathway to sustain visibility, while the target is not, so it would rely on the M-pathway for detection. The notion that rivalry (and conscious visibility in general) may primarily be attributed to the P-pathway received support from several studies^37, 38, 44, 45, 51–53^. For example, Carlson and He^51^ found that when local conflict between two orthogonally moving gratings is minimized by a grid superimposed binocularly on top, the motion from the two gratings was always *integrated*, i.e., the gratings combined into a drifting grid with the vector sum of the two motion directions dominating the percept. Similarly, for briefly presented random dot arrays, Van de Grind *et al*.^37^ reported transparency, i.e., form and motion integration. Based on these and other findings, He *et al*. (2005) concluded that, rather than motion per se, it is the conflict of local spatial elements that leads to rivalry, which can be then viewed as a property of the P-pathway. In fact, in early work on the M and P pathway disparity, Livingstone and Hubel (1988) suggested that the “magno system is not capable of sustained scrutiny” and viewed it as more primitive, while “the parvo system seems to be important for analyzing the scene in much greater and more leisurely detail” (p. 240). In this vein, the mask would produce optimal suppression whenever it satisfies the specific criteria for P-pathway activation. This explanation fits the current results reasonably well, as P-bias toward the lower temporal frequency (speed, in our case) led to the most perceptually stable mask.

It is tempting to suggest that the current results can be explained in terms of the P-bias of the mask and M-bias of the target: features with a known bias toward the P-pathway, such as low velocity, would contribute to a more effective mask, while the target possessing the features with M-bias, such as high velocity, would be detected faster. However, there are three important caveats to this explanation. First, Yang and Blake (2012) found that high spatial frequency targets were more easily detected, while a low spatial frequency mask was more effective in suppressing low spatial frequency targets. Both of these findings contradict the proposed target and mask correspondence to M- and P-pathways, respectively. Second, the use of color in the mask, which could potentially bias the mask further toward the P-pathway, was found to be non-essential in masking motion^26^. Finally, there are reports of rivalry occurring between dynamic stimuli, even when local spatial conflicts are minimized^54^. With regards to the last study, the authors found that the temporal characteristics of the two stimuli have to diverge sufficiently in order to rival, counter to He *et al*.’s (2005) proposal that rivalry is primarily a property of P-pathway.

However, the possibility that the M-pathway ‘specializes’ in detection may still hold. The magno-pathway feed-forward signal reaches area MT milliseconds after V1, considerably earlier than along the parvo/ventral stream [mean neuronal response latencies V1 = 72 ms; MT = 76 ms; Temporal> 120ms^55^]. This fast magno/dorsal feed-forward sweep was suggested to ensure early top-down feedback to lower visual areas even before the parvo/ventral signal reaches V1^49, 56^. This ‘private line’ of motion perception, and prioritization of movement detection over object identification, is hypothesized to possess an evolutionary benefit^57^.

It is notable that the issue of the stability of the conscious percept (here operationalized as the effectiveness of the mask) has been largely ignored in the interocular suppression literature. This is despite the fact that the biases of an effective mask could affect the range of features that are effectively suppressed, as demonstrated by Yang & Blake^24^. Why such biases arise is an open question. While it may be easy to explain the target detectability in terms of its salience^40^, a ready explanation does not seem to be available when it comes to perceptual stability of the dominant image. The fact that matching the speed of a highly salient stimulus does not make the mask more effective seems to suggest that *a salient mask is a less effective mask*.

To summarize, neither a salient nor a matching mask was effective in suppressing a moving target; rather, the mask moving at a low velocity was the most effective, regardless of the dynamic characteristics of the target. A more salient target, however, was detected faster regardless of the mask. This study therefore reveals a remarkable independence of mask features from those of the target, and raises the question of what drives the perceptual stability of conscious content.

## Methods

### Apparatus

Stimuli were presented against a black background on a 22-in. Samsung 2233RZ LCD monitor with a resolution of 1680×1050 pixels and a refresh rate of 120 Hz. Throughout the experiment, a white frame (subtending 5°x5° of visual angle) remained on the screen to facilitate fusion of the two images, which the subject viewed via a custom-made four-mirror stereoscope mounted on a chinrest.

The stimuli were presented and responses recorded using the PsychoPy stimulus presentation package for Python^58, 59^.

### Stimuli

The mask and target were similar to that used by Moors *et al*. (2014). The target stimulus was a continuously moving gray disk with a diameter of 0.46° of visual angle (Fig. 1A,B). The pattern of motion of the target was an independent variable in Experiment 1 (either linear or rotational), while always being rotational in Experiment 2. For rotational motion, the target moved clockwise or counter-clockwise at a distance of either 1.2° or 1.4° of visual angle away from the fixation point. It could start its motion at an angular distance of 0, 120, or 240° along the trajectory. For linear motion, the target moved left or right, either above or below the fixation point at vertical distances of 1.1° of visual angle away from the horizontal midline of the viewing frame. The linear target could start its motion at –1°, 0°, or 1° of visual angle relative to the vertical midline. Once the target reached the edge, it reset its path on the other side of the trajectory. For both rotational and linear motion, the trajectory location, starting position, and direction of motion were randomized.

The mask comprised 248 squares, colored red, blue, green, and yellow, and 0.41°^2^ in area, matching the surface area of the target (Fig. 1C,D). The mask moved either linearly or rotationally in Experiments 1, while always moving rotationally in Experiment 2. For rotational motion, half of the mask elements moved clockwise, and the other half counter-clockwise at a velocity measured in visual degrees per second (so the mask elements closer to the center, with smaller orbital radii, were moving at higher angular speeds). For linear motion, the mask elements moved left, right, up, or down, with a quarter moving in each direction.

Across all conditions, all targets and mask elements moved within a virtual circle with a 5° of visual angle diameter and were refreshed simultaneously at 60 Hz. A white fixation dot, 0.2° of visual angle in diameter, was positioned in the center of the frame, and the participants were asked to maintain fixation on it at all times.

### Procedure

The work was carried out in accordance with the Code of Ethics of the World Medical Association (Declaration of Helsinki) and approved by the Duke-NUS Institutional Review Board. Informed consent was obtained from all participants involved in the study.

The dependent variable was the time it took for the participant to detect the target. The target was presented to the non-dominant eye for 3 s, during which its contrast rose from 0 to 1.5 times the threshold value (threshold reached at 2 s). The mask was presented to the dominant eye, with the dominance determined by the individual threshold test (see below). The onset of the target was jittered (0–500ms). The participants were asked to indicate the direction of the moving target by pressing a key as soon as they detected the target. Pilot experiments showed this procedure to be efficient and comparable to 2AFC-based threshold calculation for each condition (see Supplemental Materials).

Prior to the main experiment, individual thresholds were calculated based on two 2-up-1-down staircases with starting contrasts of 0.1 and 1, where the threshold was the contrast (of the target and the mask, see below) at which target detection took less than 2 s. For the purposes of the staircases, the ‘correct’ response was the participant’s detection between 0.5 and 2 s; no-response trials and shorter or longer responses were classified as ‘incorrect’. Because the maximum contrast was varied depending on the participant’s response within this time frame, the resulting threshold value ensured that target detection took place neither too early nor too late for any given subject. Each staircase terminated after eight reversals, with the last six used for the average threshold estimates. One such threshold was obtained for each eye, with the higher threshold considered to belong to the non-dominant eye, and taken for the main experiment. The threshold was used to ensure variability in participant’s suppression times to allow detection of meaningful differences among experimental conditions.

Due to the strength of the mask, approximately a half of the participants found it difficult to detect the target even at the target contrast threshold of 1, so the mask contrast was allowed to vary. As such, the threshold values could range from zero to 2, where the value range of 1–2 meant that the mask contrast was lowered (e.g., a threshold of 1.1 meant that, in the main experiment, the target was ramped up to full contrast at 2 s, while the mask contrast was fixed to 0.9; therefore the difference between the thresholds, e.g., 1.1 and 1.2 was only in mask contrast).

Each experiment took 35–60 min to complete (including the preliminary threshold measurement).

### Analyses

All analyses were carried out using the R statistical programming environment^59^. Package *lme4* was used to run mixed models with the participant as a random variable^60^. This is an improvement over traditional approaches (such as repeated-measure ANOVA) to handling random effects, due to mixed models’ resistance to missing data and homoscedasticity violations, as well as increased statistical power^61, 62^. The *t*-values are reported for individual predictors and interaction terms. However, due to the mathematical ambiguity in calculating degrees of freedom for mixed models^61^, the *df* terms are not provided. We include approximate *p*-values calculated based on the *t*-statistic and normal distribution function using *lmerTest* R package. The marginal effects were evaluated with Bayesian statistics using *BayesFactor* R package^63^. Per convention, a Bayes Factor (BF) between 1 and 10 was considered inconclusive in model comparison; BF < 1 as indicating preference toward the denominator model; and BF> 10 toward the nominator model. The *ggplot2* package was used to visualize the data^64^.

### Participants

For Experiment 1, thirteen participants were recruited from the National University of Singapore student body and compensated $10 for their time. Two participants were excluded due to incomplete data. Data from nine female and two male participants were included in the analyses (age mean = 24.2, SD = 2.6). All had normal or corrected-to-normal vision.

For Experiment 2, twelve participants (seven male) were recruited for the study, including two of the authors (EA and PJH).

For Experiment 3, fifteen naïve participants (five male) were recruited for the study. All had normal or corrected-to-normal vision.

### Design

In Experiment 1, the target always moved at a speed of 3°/s, while the mask speed varied randomly among 1, 2, 3, 5, and 8°/s. Both the target and the mask elements could move either linearly or rotationally. As a result, there were 2 target motion patterns × 2 mask motion patterns × 5 mask speeds = 20 conditions, with 18 trials in each. Four combinations of target and mask motion patterns were blocked, with the order of blocks counterbalanced across participants. Each block contained 90 non-blank trials (18 trials per mask speed) and 18 blank trials, to discourage random responses.

In Experiment 2, the target always moved at speeds of either 1 or 5°/s, while the mask speed varied randomly among 0.3, 1, 2, 3, 5, and 8°/s. As a result, there were 2 target speeds × 6 mask speeds = 12 conditions, with 24 trials in each.

In Experiment 3, the target and mask motion pattern and speeds were the same as in Experiment 2, but the targets reversed their direction five times in three seconds (i.e., once every .6 s), regardless of their speed.

### Data Availability Statement

The scripts for running and analyzing the experiments were made available at https://github.com/egorananyev/supreff. Data will be provided upon request.

## Electronic supplementary material

Supplementary Information
