# Supplementary material for: Separate requirements for detection and perceptual stability of motion in interocular suppression
Source: Sci Rep. 2017 Aug 3;7:7230. doi: 10.1038/s41598-017-07805-5 (PMC5543169; doi:10.1038/s41598-017-07805-5)
Supplement: Supplementary file 1 — Supplementary Information [file 41598_2017_7805_MOESM1_ESM.pdf]

Title

Separate requirements for detection and perceptual stability of motion in interocular suppression

Authors
Egor Ananyev[1] egor.ananyev@gmail.com
Trevor B. Penney[1,2] penney@nus.edu.sg
Po-Jang (Brown) Hsieh[3] pojang.hsieh@duke-nus.edu.sg

Affiliations
[1]Department of Psychology, National University of Singapore
[2]LSI Programme in Neurobiology and Aging, National University of Singapore
[3]Neuroscience and Behavioral Disorders Program, Duke-NUS Graduate Medical School

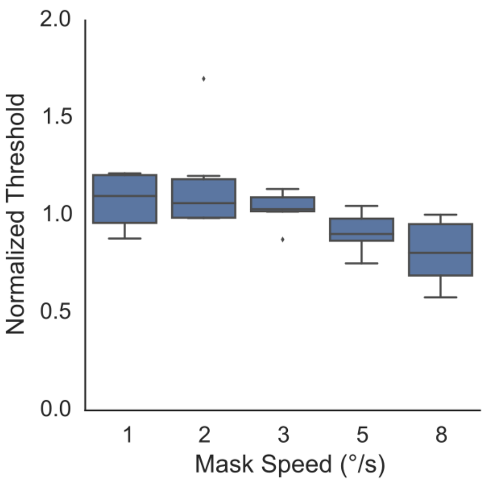

Supplementary Figure S1. The results of a contrast threshold version of the linear target / linear mask condition in Experiment 1. Higher mask speeds led to lower contrast thresholds (b = −0.008, t = −2.875, p = 0.009), consistent with the detection time-based measurements. A quadratic non-linear effect was significant (F[1,3] = 10.677, p = 0.047).

A: Low-threshold participants

B: High-threshold participants

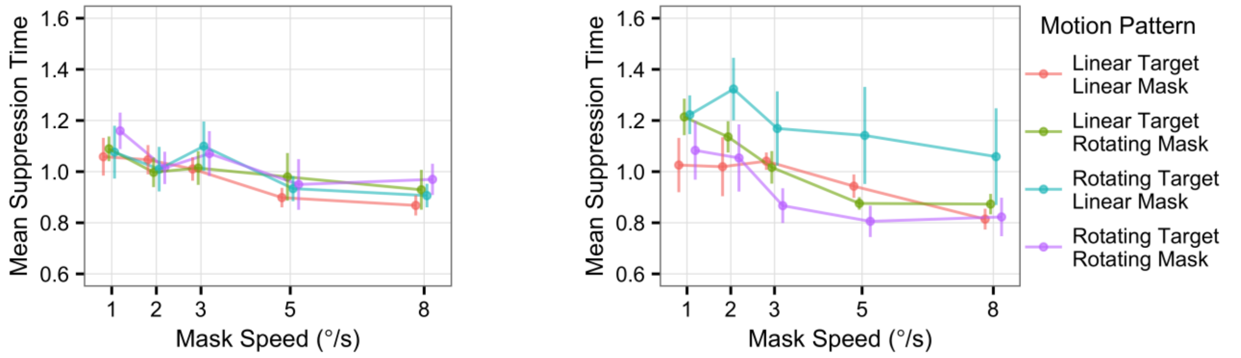

Supplementary Figure S2. The relationship between the individual contrast thresholds and the size of the motion pattern and speed effects on suppression times. Results for five low-threshold (A; mean = 0.89) and five high-threshold (B; mean = 1.73) participants; median split with the median-threshold participant excluded (1.37). The error bars reflect standard errors.

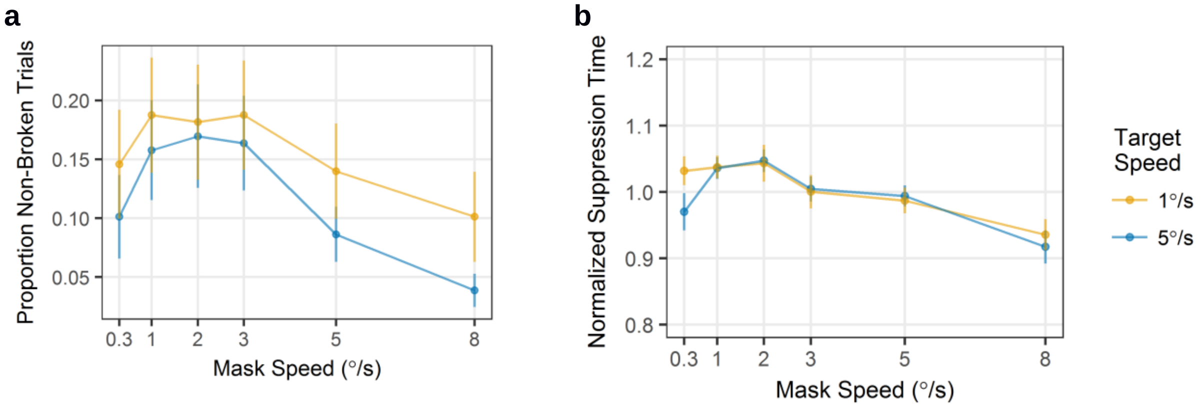

Supplementary Figure S3. The results of an experiment when the number of iterations a target travelled its path was the same for both target speeds (1°/s and 5°/s). The differences between this experiment and Experiment 3 were that (a) the target travelled a linear path, and (b) instead of reversing direction upon completing its path, it reset to original location and repeated it, as linear targets did in Experiment 1. (**a**) The proportion of no-response trials as a function of target and mask speeds. As in Experiment 3, there was a significant reduction in non-broken trials for faster targets ($b = -2.176$, $t = -3.42$, $p = 0.001$, $BF = 50.7$) and faster masks ($b = -1.277$, $t = -2.63$, $p = 0.009$). Due to the quadratic nature of the relationship, the latter effect did not reach significance in Bayesian terms ($BF = 5.08$). The interaction was not significant ($p > 0.499$). (**b**) Normalized suppression times as a function of target and mask speeds. There was no significant effect of target speed on the time to detect the target ($b = -0.003$, $t = -1.49$, $p = 0.135$, $BF = 0.043$), but a significant effect of mask speed ($b = -0.015$, $t = -9.35$, $p < 0.001$, $BF > 10^{16}$), with a marginally significant interaction ($b = 0.002$, $t = 2.72$, $p = 0.006$, $BF = 1.078$).

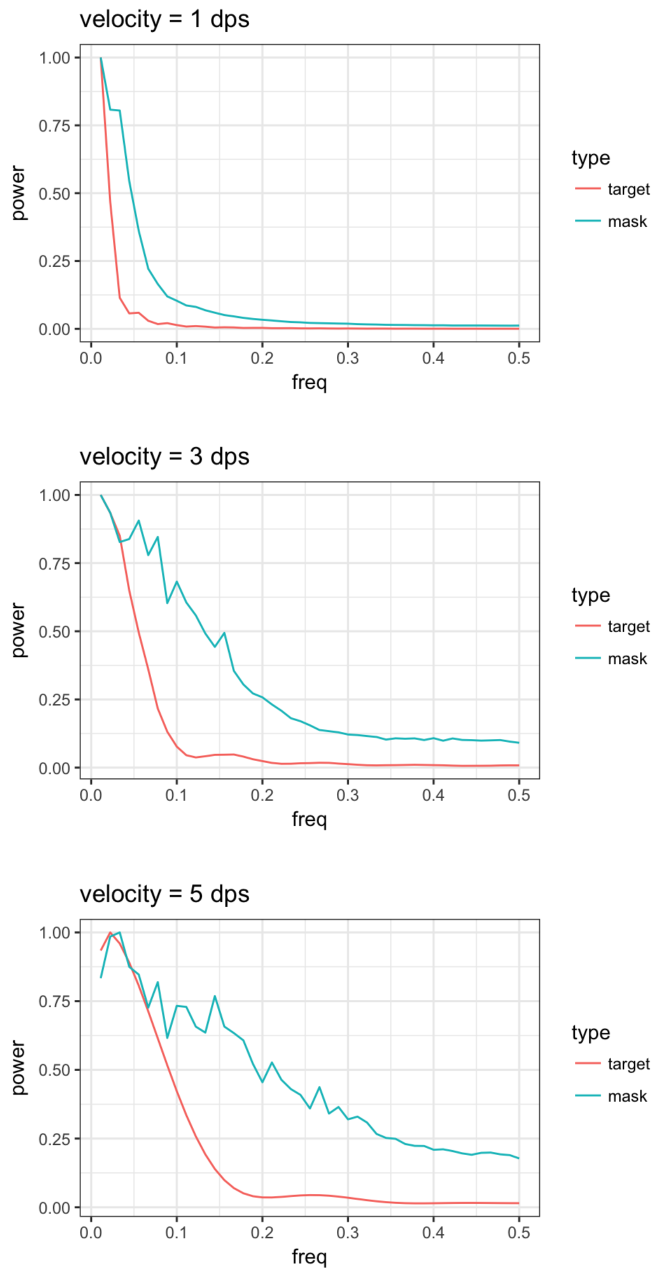

Supplementary Figure S4. The normalized power spectra of the target and mask at different velocities as a function of the temporal frequency (averaged across all pixels in the respective display). Increasing the velocity of both the target and the mask had a similar effect: the frequency profile was widened to include the higher end of the spectrum. However, as matching the temporal frequencies of the target and mask did not lead to more effective suppression (just as matching the velocities did not), no feature-selective suppression was observed.
